# Chronic Pharyngitis Accidentally, Yet Successfully Treated by a New Process of Cauterization

**Published:** 1880-11-20

**Authors:** J. S. Stidham

**Affiliations:** Georgia


### CHRONIC PHARYNGITIS ACCIDENTALLY, YET SUCCESSFULLY TREATED BY A NEW PROCESS OF CAUTERIZATION.

BY J. S. STIDHAM, M.D., OF GEORGIA.

Last spring my esteemed friend, Dr. G——, had occasion to cauterize the pharynx in a case of chronic pharyngitis. After preparing his stick of caustic in a quill, the usual procedure began. He had made only one or two slight strokes when the pharynx and surrounding parts suddenly took on a spasmodic contraction, nipped off the stick of caustic near the quill, and gave it a good send down the œsophagus.

After an examination of his unarmed quill the Doctor says his diagnosis was "a partial cauterization of the pharynx, and patient with about 50 grains of caustic in the stomach."

*Doctor.* "How do you feel?"

*Patient* (not aware of present state of affairs). "Very well, except my throat burns me a little."

Ordered cup of warm water, put in 20 grains ipecac, and sent it down after his silver. The Doctor took his seat for a short time, and we will suppose *felt* a great deal worse than his patient.

*Doctor.* "Do you feel sick at your stomach?"

*Patient.* "No, sir, I feel very well."

Ordered more warm water, and with 30 grains ipecac turned it in on his silver. In due time the stomach was relieved of his cauterizing agent. The Doctor, relieved of his nervous diathesis, and patient dismissed.

Now the Doctor claims a fine application of his caustic, just as it left his quill, and then a solution of 50 grains to the pint of water returned over the same surface. Hence, "a successful treatment."

The Dr. don't propose to try this treatment again (unless by accident), neither is he inclined to suggest it to the profession; nevertheless he declares his patient, who has been troubling him the last six months for gargles, washes, etc., is no longer a pest, but is cured of her "throat disease."

